# Viral Gastroenteritis: Sickness Symptoms and Behavioral Responses

**DOI:** 10.1128/mbio.03567-22

**Published:** 2023-03-28

**Authors:** Arash Hellysaz, Magdalena Neijd, Timo Vesikari, Lennart Svensson, Marie Hagbom

**Affiliations:** a Division of Molecular Virology, Department of Clinical and Experimental Medicine, Linköping University, Linköping, Sweden; b Nordic Research Network Oy, Tampere, Finland; c Division of Infectious Diseases, Department of Medicine, Karolinska Institute, Stockholm, Sweden; Albert Einstein College of Medicine

**Keywords:** rotavirus, norovirus, sickness symptoms, brain, hypothalamus, behavioral responses, noroviruses

## Abstract

Viral infections have a major impact on physiology and behavior. The clinical symptoms of human rotavirus and norovirus infection are primarily diarrhea, fever, and vomiting, but several other sickness symptoms, such as nausea, loss of appetite, and stress response are never or rarely discussed. These physiological and behavioral changes can be considered as having evolved to reduce the spread of the pathogen and increase the chances of survival of the individual as well as the collective. The mechanisms underlying several sickness symptoms have been shown to be orchestrated by the brain, specifically, the hypothalamus. In this perspective, we have described how the central nervous system contributes to the mechanisms underlying the sickness symptoms and behaviors of these infections. Based on published findings, we propose a mechanistic model depicting the role of the brain in fever, nausea, vomiting, cortisol-induced stress, and loss of appetite.

## PERSPECTIVE

Rotavirus and norovirus are two major causes of acute gastroenteritis and are characterized by vomiting, fever, and diarrhea. While norovirus is quite common in adults who can communicate their illness experience very well, rotavirus occurs mainly in children below the age of 5, which have difficulty conveying their illness. Our knowledge of how rotavirus causes illness has therefore mainly been obtained from investigating the mechanisms of diarrhea (1–6). Although these observations are compelling and have provided important mechanistic information on rotavirus diarrhea, many aspects of rotavirus illness remain unexplored. Only a few studies have investigated the gut-brain communication underlying vomiting (7, 8) and intestinal motility (9, 10) following rotavirus infection. Nonetheless, our knowledge of how the gut communicates with the central nervous system (CNS) to initiate sickness symptoms (11, 12) remains scarce. For human norovirus, our knowledge is limited due to the lack of a small animal model.

**FIG 1:**
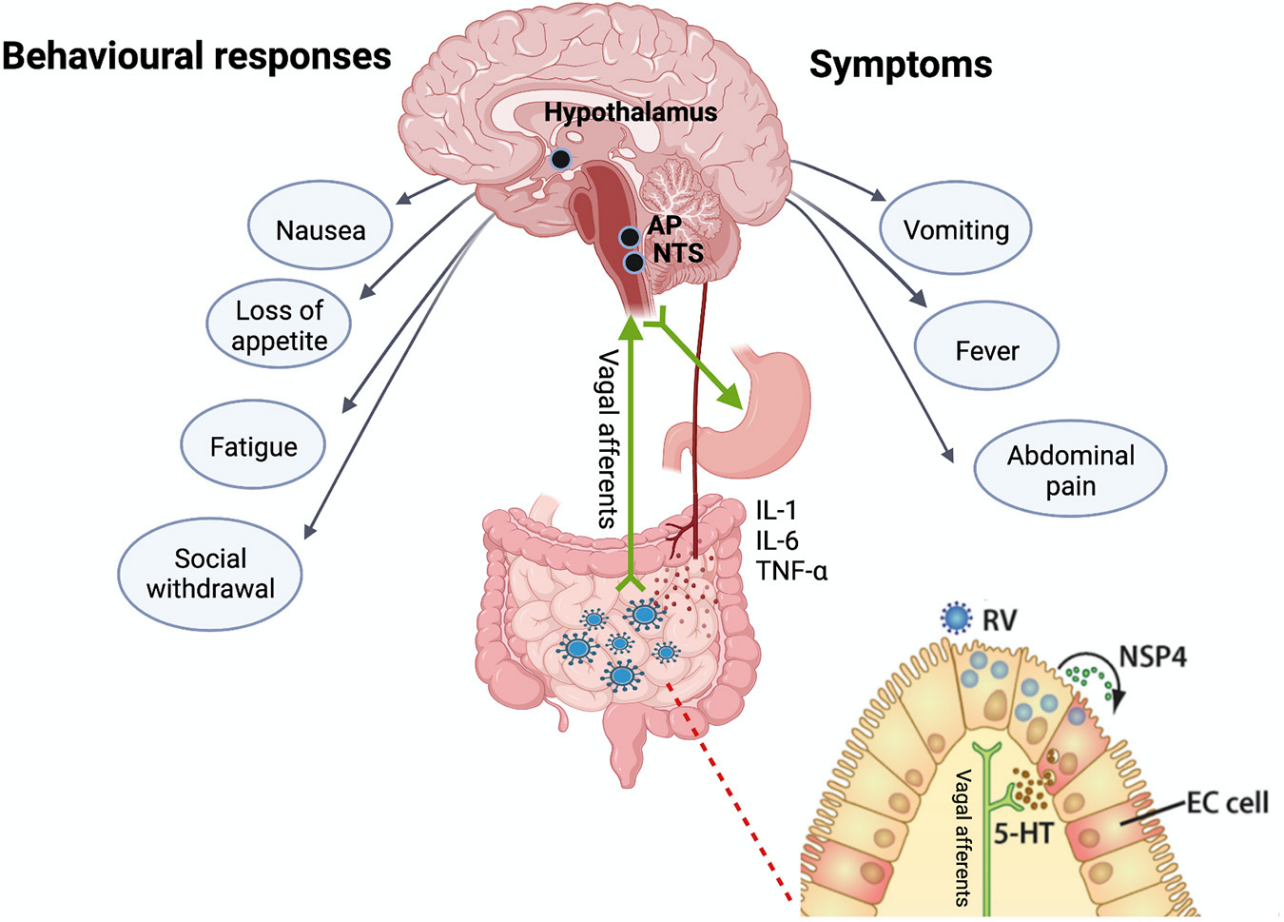
Behavior and symptom responses following rotavirus and norovirus infections. After infection, the host initiates a series of defensive responses to combat the assault. The clinical illness of rotavirus and norovirus infection can, apart from diarrhea and vomiting, include nausea, stomach pain, cramp, fever, fatigue, loss of appetite, irritability, and social withdrawal, collectively known as sickness symptoms (16–20). Such sickness symptoms can be divided into physiological and behavioral responses. Rotavirus and/or the rotavirus enterotoxin NSP4 (green dots) (8) and norovirus (49) recognize yet unknown receptors on enterochromaffin (EC) cells. These cells then respond by the release of serotonin (5-HT, brown dots), which can activate the enteric nervous system and vagal afferents that project signals to the NTS and AP, structures of the vomiting center (8). Efferent vagal signaling from the vomiting center stimulates a nerve-muscle vomiting reflex in the stomach. Vomiting and behavioral responses, such as nausea and loss of appetite, probably serve as signals for avoidance to prevent future ingestion of the pathogen. Body temperature is regulated by the hypothalamus which functions as a thermostat. Fever is initiated following infection of enterocytes in the small intestine resulting in a low-grade inflammatory response involving locally produced IL-1, IL-6, and TNF-α (red dots), which reach the thermoregulatory center of the hypothalamus. In brief, the blood-circulating cytokines (red dots) acts on the endothelium of the brain to augment the synthesis of cyclooxygenase 2, the enzyme responsible for oxidizing arachidonic acid to produce prostaglandin E2 (PGE2). The hypothalamus then releases PGE2 from the preoptic area which increases the body temperature and triggers vasoconstriction and relocating blood to internal organs from the peripheries to limit heat loss from the skin surface (41, 62). The activated immune system discourages nutrient intake and promotes lower nutrient release in the bloodstream, thus restricting systemic nutrient availability. Nutrient restriction may limit pathogen replication and seems to be prioritized at the expense of organ starvation. These changes in physiology and behavior help the host fight the infection, increase survival, and reduce pathogen spreading. RV, rotavirus; NSP4, rotavirus toxin, nonstructiral protein 4; 5-HT, serotonin; EC cell, enterochromaffin cell; IL, interleukin; TNF-α, tumor necrosis factor alpha; AP, area postrema; NTS, nucleus of the solitary tract. The illustration is modified from (2, 4) and created by BioRender.com.

In this perspective, we discuss the mechanisms of certain sickness behaviors and symptoms coordinated by the brain and associated with rotavirus and norovirus infections (Fig. 1).

## SICKNESS SYMPTOMS CHANGE PHYSIOLOGY AND BEHAVIOR

In addition to vomiting, fever, and diarrhea, the clinical illness of rotavirus and norovirus can include nausea, headache, abdominal discomfort, myalgias, fever, fatigue, and loss of appetite (13–15), collectively known as sickness symptoms (16–20) (Fig. 1). These changes in physiology and behavior can be considered as having evolved to help the organism to fight pathogens, increase survival, and reduce spreading (21). Although the main defense is coordinated by the immune system, most sickness symptoms are orchestrated by the brain (22) and occur at different time points of the disease to achieve different goals. For instance, rotavirus infection can debut with sickness symptoms before the onset of diarrhea (23, 24), and cause altered brain activities associated with infection-induced stress response (12). Another early sickness characteristic is illustrated by norovirus-induced vomiting, which often occurs very suddenly and can occur before the onset of diarrhea (13). These are examples of early gut-brain communication driving sickness symptoms.

## THE HYPOTHALAMUS IS A MAJOR HUB FOR CONTROLLING SICKNESS SYMPTOMS AND BEHAVIORS

The gut-brain axis is a bidirectional communication between the central and the enteric nervous systems (CNS and ENS, respectively) (25), which provide the means to maintain gastrointestinal homeostasis, but also coordinate food intake and energy expenditure (26, 27). For accurate output, the brain must monitor, integrate, and link gut functions with the emotional, cognitive, and behavioral centers of the brain. The connection of the CNS with the ENS occurs through the sensory and autonomic nervous systems, including the endocrine system. The sensory systems provide afferent signals arising from the gut lumen and the intestinal wall, through signals within the ENS and their connections to vagal pathways which directly connect the gut to brain areas such as the nucleus of the solitary tract (NTS) and the bed nucleus of the stria terminalis (BNST) (28). Information can also reach the brain through the blood system, e.g., the interleukin (IL)-1 β, IL-6, and tumor necrosis factor alpha (TNF-α) which can initiate fever (17). Recent studies have identified the NTS and BNST to be activated and deactivated following rotavirus infection (8, 12). Even though precise pathways between rotavirus- and norovirus-infected enteric cells and neurons of the NTS and BNST remain unknown, both areas are known to propagate peripheral sensory inputs through different nuclei of the hypothalamus to ultimately control nonfear related stress (28), appetite, and fever (20). The NTS and BNST are thereby likely to be involved in several of the sickness symptoms caused by rotavirus and norovirus. These findings provide evidence for the role of the gut–brain axis in the mechanisms of the sickness symptoms of human rotavirus and norovirus infections.

## ANOREXIC BEHAVIORS CAUSED BY ROTAVIRUS AND NOROVIRUS INFECTIONS

Norovirus (15) and probably rotavirus infection lead to loss of appetite which influences our physiology and body hemostasis with changes in systemic metabolism through the secretion of cytokines and altered endocrine control (29). Given the high energy requirements to sustain immune responses and healing processes, it is intriguing that reduced appetite and anorexic behaviors are common sickness symptoms (30). Despite the immune system’s increased demand for nutrients, the activated immune system seems to discourage nutrient intake and promote metabolic changes that lower nutrient release in the bloodstream, thus restricting systemic nutrient availability (31). Restriction of nutrient availability to limit pathogen replication seems to be prioritized at the expense of organ starvation. This is particularly true for glucose, which is a nutrient that viruses use to promote their replication (32). As a result, infection promotes a form of metabolism that is normally associated with fasting (31), resulting in lost appetite. Furthermore, a preoptic neuronal population appears to control appetite during sickness (20). Both rotavirus and norovirus stimulate the release of TNF-α (33, 34), a cytokine that promotes the release of leptin (18), which mainly binds to leptin receptors in the proopiomelanocortin (POMC) neurons in the arcuate nucleus of the hypothalamus (ANH) and suppresses appetite (35). Intriguingly, leptin-sensitive POMC neurons are also present in the NTS (36) and hypothalamic POMC neurons have been identified to innervate several brain areas, including the preoptic regions (37). While the immune system seems to *promote* a metabolic state similar to fasting, the brain and particularly the hypothalamus, seem to *drive* the adaptive physiological and behavioral changes necessary for the suppression of appetite during viral infections.

## HYPOTHALAMIC REGULATION OF THERMOGENESIS DURING ROTAVIRUS AND NOROVIRUS INFECTION

Thermoregulation and central control of body temperature are mainly governed by the hypothalamus (38). A not uncommon symptom during rotavirus and norovirus infections is fever (13, 15, 39), a hallmark of infectious diseases. It is generated by the concerted action of autonomic responses, including decreased thermogenesis, sweating, peripheral vasoconstriction, reduced heat loss, and shivering. Fever is considered beneficial because an elevated body temperature enhances the activity of the immune cells and impairs the replication of viruses. Human rotavirus infection has been shown to stimulate the release of IL-1β, IL-2, IL-4, IL-6, IL-8, IL-10, IL-12, interferon-gamma (IFN-γ), and TNF-α (33). Accordingly, children with fever have been found to have significantly elevated levels of serum IL-6 compared with control children, and those with fever and more episodes of diarrhea had significantly higher levels of TNF-α than those without fever and with fewer episodes of diarrhea (33). These markers of innate immune response occur early in infection and contribute to the pathogenesis and to sickness behavior. Indeed IL-1β, TNF-α, and IL-6 stimulate the endothelium of the brain to produce prostaglandins (PG), such as PGE2 which result in increased body temperature (40). This leads to heat-promoting events, such as vasoconstriction and shivering, as well as changes in the metabolic rate of cells in the periphery, such as induction of thermogenesis in brown adipose tissue (16, 41, 42). Similar to rotavirus, norovirus induces elevation of IL-2, IL-6, and TNF-α (34), resulting in fever response with both viruses (13–15).

## NAUSEA AND VOMITING ARE CNS-COORDINATED SICKNESS SYMPTOMS OF ROTAVIRUS AND NOROVIRUS INFECTIONS

Nausea and vomiting are hallmarks of the sickness symptoms of rotavirus and particularly norovirus, which causes the winter vomiting disease. While being common symptoms, surprisingly little information is available on how these viruses trigger nausea and vomiting (2, 4, 8, 12, 43, 44). The fact that norovirus can rapidly induce nausea and vomiting in the absence of diarrhea and with an incubation as short as 10 h (13), strongly argues for nervous control of nausea and vomiting. Further, some characteristic physiological changes occurring before vomiting include increased blood pressure, tachycardia, sweating, salivation, and vasoconstriction, which are mediated by the autonomic nervous system (ANS) (45).

Enterochromaffin (EC) cells constitute the largest population of epithelial enteroendocrine cells and are sensory cells that release serotonin (5-hydroxytryptamine [5-HT]) upon stimulation (46). Released serotonin then stimulates vagal afferents, the major peripheral emetic nerves that participate in vomiting, in response to emetic stimuli in the gastrointestinal tract (47), including rotavirus (4, 8) and probably also norovirus. This is further supported by Xie and coworkers’ (48) reporting that distinct brainstem circuits drive enterotoxin-induced nausea and retching through nerve signaling in response to serotonin release from EC cells. In addition, rotavirus has been shown to stimulate the release of serotonin from human EC cells and activate structures in the CNS involved in nausea and vomiting (8). Likewise, Jung and coworkers (44) suggested that serotonin release from EC cells into the gut submucosa might occur early following porcine epidemic diarrhea virus (PEDV) infection. They suggested that serotonin and vagal afferent neurons might be involved in the mechanism of vomiting in PEDV-infected piglets. Moreover, Green and coworkers have showed that human norovirus infects EC cells in the small intestine (49), and Chang-Graham et al. (50) have reported that rotavirus can stimulate the release of serotonin from human enteroids. In accordance with these findings, ondansetron, a 5-HT_3_ receptor antagonist, has been found to reduce rotavirus symptoms in children (43). Through transcriptomic analysis of human enteroids infected with human norovirus, Mirabelli and coworkers found (51) upregulation of certain hormones and neurotransmitter signal transduction pathways.

Based on the above findings, the key sites involved in the mediation of vomiting following viral gut infection are as follows: (i) the sensory epithelial EC cells, which are present in the mucosa of the gastrointestinal tract; (ii) the enteric nervous system and the vagus nerve; (iii) the brainstem dorsal vagal complex emetic nuclei such as the area postrema (AP), the NTS, and the dorsal motor nucleus of the vagus (DMV). However, further investigations into the underlying neuroanatomical and neurochemical mechanisms are required to translate these observations into a mechanistic model of rotavirus and norovirus vomiting and nausea (2, 4).

## GUT HOMEOSTASIS AND INTESTINAL MOTILITY ARE REGULATED BY THE CNS

Although the ENS can work independently of CNS and control most of the intestinal functions, CNS can trough the autonomic nervous system modulate intestinal motility and gut homeostasis (52). The inhibitory and excitatory effects on the small intestine through the autonomic sympathetic and the parasympathetic systems are well established (53, 54). Under homeostasis, there is a balance of the two opposing systems, and balance disruption by either up- or downregulation in either system can disrupt proper motility control and lead to either diarrhea or constipation. It has been shown that rotavirus infection disrupts the autonomic balance by inhibitory effect on the noradrenergic sympathetic nervous system in ileum and increase intestinal transit before the onset of diarrhea (12).

## HUMAN ROTAVIRUS INFECTION LEADS TO CORTISOL-INDUCED STRESS FROM THE HYPOTHALAMUS

Infections are associated with stress, as indicated by the concomitant release of the steroid hormone cortisol (55). When the body senses threats such as an infection, this stressor will affect several systems, including neurons of the BNST. The BNST serves as a relay center between limbic cognitive centers and nuclei involved in the processing of reward, stress, and anxiety, and has connections with other limbic structures, as well as with hypothalamic and brainstem nuclei involved in the control of autonomic, neuroendocrine, and behavioral responses (56). Moreover, it receives ascending information regarding systemic stressors, such as blood pressure and heart rate. The BNST has direct input into the hypothalamus which regulates the production and secretion of cortisol in the hypothalamus-pituitary-adrenal axis (HPA) (57). Specific neurons in the hypothalamus releases the neuropeptide corticotrophin-releasing hormone (CRH), which is transported through the capillary plexus of the hypothalamo-hypophyseal portal system to reach the pituitary gland (58). There, CRH affects pituitary corticotropes to release blood-circulating hormones that signal to the adrenal cortex in the kidney to secrete cortisol. Blood circulating cortisol triggers a stress response, including inhibition of the production of IFN-α, IL-12, and TNF-α. It has been shown that rotavirus infection causes deactivation of the BNST in mice (12) and that infection can increase stress-related cortisol levels in humans (59, 60). Neville and coworkers (60) noted that children with rotavirus gastroenteritis constitute to exhibit significant stress with cortisol levels well above control levels. Further, a systemic review of several infectious diseases by Rezai and coworkers (61) noted most interestingly that children with severe gastroenteritis had the second highest level of cortisol in the blood. This indicates that symptomatic rotavirus infection causes a significant cortisol stress response, and further strengthens the role of the hypothalamus in rotavirus-induced illness (12).

## SUMMARY

In this perspective, we have discussed how the central nervous system contributes to the mechanisms underlying the sickness symptoms and behaviors during virus infection of the gut. To further increase understanding, new approaches such as organoid models and three-dimensional (3D) light sheet microscopy of gut and brain may be of benefit.
